# Fibrinolysis and Proliferative Endarteritis: Two Related Processes in Chronic Infections? The Model of the Blood-Borne Pathogen *Dirofilaria immitis*

**DOI:** 10.1371/journal.pone.0124445

**Published:** 2015-04-13

**Authors:** Javier González-Miguel, Rodrigo Morchón, Mar Siles-Lucas, Fernando Simón

**Affiliations:** 1 Laboratory of Parasitology, Faculty of Pharmacy, Institute of Biomedical Research of Salamanca (IBSAL) and University of Salamanca, Salamanca, Spain; 2 Laboratory of Parasitology, IRNASA, CSIC, Salamanca, Spain; Institute of Biochemistry and Biotechnology, TAIWAN

## Abstract

The interaction between blood-borne pathogens and fibrinolysis is one of the most important mechanisms that mediate invasion and the establishment of infectious agents in their hosts. However, overproduction of plasmin (final product of the route) has been related in other contexts to proliferation and migration of the arterial wall cells and degradation of the extracellular matrix. We have recently identified fibrinolysis-activating antigens from *Dirofilaria immitis*, a blood-borne parasite whose key pathological event (proliferative endarteritis) is produced by similar mechanisms to those indicated above. The objective of this work is to study how two of this antigens [actin (ACT) and fructose-bisphosphate aldolase (FBAL)] highly conserved in pathogens, activate fibrinolysis and to establish a relationship between this activation and the development of proliferative endarteritis during cardiopulmonary dirofilariasis. We demonstrate that both proteins bind plasminogen, enhance plasmin generation, stimulate the expression of the fibrinolytic activators tPA and uPA in endothelial cell cultures and are located on the surface of the worm in contact with the host’s blood. ELISA, western blot and immunofluorescence techniques were employed for this purpose. Additionally, the implication of lysine residues in this interaction was analyzed by bioinformatics. The involvement of plasmin generated by the ACT/FBAL and plasminogen binding in cell proliferation and migration, and degradation of the extracellular matrix were shown in an “in vitro” model of endothelial and smooth muscle cells in culture. The obtained results indicate that ACT and FBAL from *D*. *immitis* activate fibrinolysis, which could be used by the parasite like a survival mechanism to avoid the clot formation. However, long-term overproduction of plasmin can trigger pathological events similar to those described in the emergence of proliferative endarteritis. Due to the high degree of evolutionary conservation of these antigens, similar processes may occur in other blood-borne pathogens.

## Introduction

The interaction between pathogens and their hosts at molecular level is the key point that mediates invasion and establishment of the infection. One of these events is the prevention of blood clotting through interaction with the hemostatic system, which is used by many blood-borne pathogens as a survival mechanism [1]. The fibrinolytic system, one of the main anticlotting mechanisms of the hemostatic system, is composed by plasminogen (PLG), an abundant component of blood and zymogen of serine protease plasmin, enzyme responsible for degrading fibrin clots. The conversion of PLG into plasmin is regulated by binding to receptors via its five kringle domains, which have affinity for lysine residues and PLG activators [tissue plasminogen activator (tPA) and urokinase-type plasminogen activator (uPA)] [2]. On the other hand, plasmin is believed to play an important role in a number of physiological and pathophysiological processes such as tissue remodeling, wound healing, angiogenesis or inflammation [3]. Overproduction of plasmin has been also linked with the proliferation and migration of human vascular cells and with the degradation of the extracellular matrix (ECM) [4–6]. In addition, plasmin is also upregulated in chronic inflammatory diseases, including atherosclerosis and arthritis [7].

Activation of fibrinolysis by pathogen antigens has been widely studied in bacteria where the expression of fibrinolytic receptors is considered an effective system for invasion and dissemination [8]. In addition, the ability to interact with the fibrinolytic system has been recently found in many eukaryotic pathogens causing parasitic infections, such as *Leishmania mexicana*, *Plasmodium falciparum*, *Fasciola hepatica*, *Schistosoma bovis* or *Onchocerca volvulus* [9]. *Dirofilaria immitis* is a zoonotic filaria responsible for canine and feline cardiopulmonary dirofilariasis and human pulmonary dirofilariasis [10]. These are chronic pathological processes that occur in the pulmonary arteries, where *D*. *immitis* adult worms survive for long periods (over 7 years) causing an inflammatory and thrombotic disease. Its key factor is the appearance of proliferative endarteritis, which results in the formation of intravascular microvilli. It has been described that this process is accompanied by an increase and migration of the cells of the arterial wall [11–14], as well as the destruction of the ECM [15]. These changes cause disorganization of the endothelium and reduction of the vascular lumen in the pulmonary arteries, with the consequent extension of pathology to the pulmonary parenchyma [16].

In previous research, we have studied the interaction between the excretory/secretory (ES) and surface antigens of *D*. *immitis* and the fibrinolytic system of the host. The ability of these antigenic complexes to bind PLG, generate plasmin in a tPA-dependent manner and to induce an overexpression of the fibrinolytic activator tPA in vascular endothelial cells in culture was demonstrated. Additionally, we have respectively identified a total of 10 and 11 PLG-binding proteins in the ES and surface extracts of the parasite, which included different isoforms of highly conserved proteins like ACT and FBAL [17,18].

Despite the fact that the pathogenic mechanisms described in the formation of intravascular microvilli during cardiopulmonary dirofilariasis are similar to those associated with the pathophysiology of plasmin, a relationship between plasmin over-production during *Dirofilaria* infection and their pathological implications has not yet been established. The aim of the present work is to study the participation of two highly conserved proteins of *D*. *immitis* (ACT and FBAL) in the activation of the fibrinolytic system of the host and to establish its relationship with the proliferative endarteritis pathogenic mechanisms in the cardiopulmonary dirofilariasis.

## Materials and Methods

### Parasite material

Adult worms of *D*. *immitis* were obtained from hearts of naturally infected dogs from Gran Canary (Canary Island, Spain) through the jugular vein using Flexible Alligator Forceps. The surgeries were carried out by veterinarians at the Veterinary Medicine Service of Las Palmas de Gran Canaria University (Canary Island, Spain) as part of a routine practice for treating animals. The pet owners were adequately informed and gave their verbal consent to the use of the samples in the study.

### RNA isolation, RT-PCR, and cloning of DiACT and DiFBAL cDNA

RNA isolation, RT-PCR and cloning of the cDNA from the selected proteins were carried out as described in detail previously [19]. Total RNA from adult worms was extracted using the NucleoSpin RNA II kit (Macherey-Nagel) according to the manufacturer’s instructions. Then, first-strand cDNA was synthesized from *D*. *immtis* adults worms RNA using the first-strand cDNA synthesis kit (Roche) as recommended by the manufacturer. The cDNA sequence of the *D*. *immitis* actin (DiACT) and fructose-bisphosphate aldolase (DiFBAL) were amplified using the following primers:

ACTFwd (+)

ACTRev (5’-CTAGAAACATTTGCGATGAACAATTG)

FBALFwd (5’-ATGACCTCTTACTCACAGTTTCTG)

FBALRev (5’-TTAGTATGCATGATTAGCAATGTAG)

The primers from DiACT were designed on the consensus sequence resulting after the alignment of ACT cDNA sequences from *Loa loa* and *Brugia malayi* (GenBank accession numbers XM_003146804.1 and XM_001894784.1 respectively). The primers from DiFBAL were designed on the consensus sequence resulting after the alignment of FBAL cDNA sequences from *O*. *volvulus*, *B*. *malayi* and *L*. *loa* (GenBank accession numbers AF155220.1, XM_001894495.1 and XM_003138767.1 respectively). PCR amplifications were performed in 1 cycle at 94°C for 5 min, 35 cycles at 94°C for 1 min, 46°C for 46 sec, and 72°C for 1 min 30 sec and 1 cycle at 72°C for 5 min. The PCR products were finally electrophoresed in an agarose gel and the bands were purified using the StrataPrep DNA Gel Extraction kit (Stratagene). The DiACT and DiFBAL PCR products were cloned into the pSC-A vector using the StrataClone PCR Cloning kit (Stratagene) following the manufacturer’s instructions. Both clones were purified with the Machery-Nagel NucleoSpin Plasmid kit.

### Expression and purification of the rDiACT and rDiFBAL proteins

PCR products containing the whole DiACT and DiFBAL coding sequences were cloned into the TOPO vector (Invitrogen) following the manufacturer’s instructions. The recombinant plasmids were transformed into the *Escherichia coli* XL1B. Transformed cells were grown in LB-agar plates with ampicillin (100 μg/ml) overnight at 37°C. Three colonies for each molecule were grown in liquid LB plus ampicillin overnight at 37°C in agitation, and cells were harvested for plasmid extraction. Extracted plasmids were digested with EcoRI to check the insert presence. Transformed vectors were used for a ligation reaction with the pDEST vector (Invitrogen). Ligation reaction was used for transformant selection as above-mentioned. Vectors containing the inserts of interest were used to transform BL-21 cells. These were grown in liquid LB plus ampicillin (100 μg/ml) overnight at 37°C in agitation. Cultures were diluted 1:10 in fresh medium and further grown until reaching an optical density (OD) of 0.5. Then, expression of the recombinant protein was induced by 0.2% L-arabinose 20% at 37°C for 4 h in agitation. The induced cells were harvested and sonicated in a buffer containing 8M urea, 100mM NaH_2_PO_4_ and 10mM Tris-Cl, pH 7.9. After a 20 min centrifugation step at 10.000 x g, the supernatant was applied to a HIS-Select Nickel Affinity Gel (Sigma) for affinity purification of the histidine-tagged rDiACT and rDiFBAL, according to the manufacturer’s instructions. Urea was eliminated by washing the column with wash buffer (100mM NaH2PO4, and 10mM Tris-Cl pH 6.3) containing decreasing concentrations of urea (from 6M to 0M). Then, the recombinant proteins were eluted with elution buffer (50mM NaH_2_PO_4_, 300mM NaCl and 250mM imidazole, pH 7.9). The eluted rDiACT and rDiFBAL were dialysed in PBS for 24 h at 4°C and stored at −80°C until use. The purity and yield of each protein obtained after purification were assessed in 12% polyacrylamide gels using Coomasie blue staining.

### Bioinformatic analyses

The deduced amino-acid sequence of rDiACT and rDiFBAL were analysed using the following bioinformatic tools: BLAST searching of the homologous sequences in the NCBI and Swissprot/Uniprot databases (http://www.ncbi.nlm.nih.gov/, http://www.uniprot.org/); analysis of conserved domains with SMART (http://smart.embl-heidelberg.de); theoretical isoelectric point (pI) and the molecular weight (MW) calculations (http://www.expasy.org/tools/pi_tool.html); prediction of transmembrane domains with the TMHMM Server v. 2.0 (http://www.cbs.dtu.dk/services/TMHMM-2.0); prediction of signal peptides with SignalP 3.0 [20] (http://www.cbs.dtu.dk/services/SignalP); search for glycosyl-phosphatidyl anchors in the sequence with the big-PI Predictor [21] (http://mendel.imp.ac.at/sat/gpi/gpi_server.html); multiple sequence alignment with ClustalW 2.1 (http://www.ebi.ac.uk/Tools/msa/clustalw2/) and prediction of the secondary structures and three-dimensional modelling with the Swiss-Model server [22] (http://swissmodel.expasy.org/). The 3-D models were visualized with the RasMol software v.2.7.5.2.

### PLG binding assays

The ability of rDiACT and rDiFBAL to act as plasminogen-binding proteins was assessed by ELISA. Following the methodology described by González-Miguel et al. (2012) multiwell microplates (Costar) were coated with 0.5 μg/well of each recombinant protein. Then, plates were blocked and incubated with increasing amounts (from 0 μg to 3 μg) of human PLG (Acris antibodies). After incubation with the corresponding antibodies and with a chromogen, binding was revealed by measuring OD at 492 nm in an Easy Reader (Bio-Rad). Between each incubation, plates were washed three times with PBS containing 0.05% Tween_20_ (PBST). In order to study the involvement of lysine residues in this binding, competition assays were performed by including 50 mM of the lysine analogue ε-aminocaproic acid (εACA) during PLG incubation [17].

### PLG activation assays

The participation of rDiACT and rDiFBAL in plasmin generation was studied following the methodology previously described [17]. In brief, 2 μg of human PLG (Acris antibodies) were incubated in PBS with 3 μg of the chromogenic substrate D-Val-Leu-Lys 4-nitroanilide dihydrochloride (Sigma) in the presence of 1 μg of each recombinant protein in a final volume of 100 μl. Activation of PLG was initiated by addition of 15 ng of t-PA (Sigma), however, plasmin generation in the absence of tPA was also analyzed. After incubation of the plates (1 h at 37°C) the hydrolysis of the chromogenic substrate, which is directly proportional to the amidolytic activity of generated plasmin was revealed by measuring absorbance at 405 nm. Each sample was analyzed in triplicate.

### Generation of an anti-rDiFBAL antiserum

Antiserum against rDiFBAL was generated by subcutaneous immunization of two New Zealand female rabbits with 3 doses of protein in 0.2% saponin solution. First dose of 1 mg at the beginning of the experiment, plus 2 doses of 500 μg 7 and 10 days later. Rabbits were sacrificed by an intravenous overdose of pentobarbital and bled 20 days after the last dose. Serum was collected, serially diluted and titred by ELISA. The reactivity and specificity of the serum was also assessed by Western blot. Animal procedures for this purpose complied with the Spanish (Real Decreto RD53/2013) and the European Union (European Directive 2010/63/EU) guidelines on animal experimentation for the protection and humane use of laboratory animals, and were conducted at the accredited Animal Experimentation Facility (Servicio de Experimentación Animal) of the University of Salamanca (Register number: PAE/SA/001). Procedures were approved by the Ethics Committee of the University of Salamanca and all efforts were made to minimize suffering. These included good practice for housing and care minimizing discomfort, distress and pain in animals.

### Immunolocalization of DiACT and DiFBAL in *D*. *immitis* adult worms

Confocal microscopy studies were carried out on adult worm sections. *D*. *immitis* adult worms were fixed in 10% buffered formalin and embedded in paraffin. For immunofluorescence, 6 μm-thick sections were placed on polylisinated slides, deparaffinised in xylene (2×8 min each), and rehydrated. Sections were then blocked with 1% BSA in PBST for 1 h at 37°C, after which they were incubated with the anti-rDiFBAL rabbit serum diluted 1:50 in blocking buffer for 1 h at 37°C. Samples were washed three times with PBST and incubated at 4°C overnight with an anti-rabbit IgG antibody conjugated to Alexa Fluor 594 (Molecular Probes) diluted 1:400 in blocking buffer containing phalloidin-Alexa Fluor 488 (Molecular Probes) diluted 1:200, which specifically binds to ACT microfilaments. The samples were then washed four times and mounted in an antifade reagent (Prolong Gold, Molecular Probes). Negative controls were carried out by serum from a non-immunized rabbit.

### Cell culture

Canine endothelial cells (CnAOEC) and canine smooth muscle cells (CnAOSMC) from Cell Applications, INC were respectively grown in canine endothelial and canine smooth muscle cell growth mediums (Cell Applications, INC). CnAOEC plates were precoated with an attachment factor solution (Cell Applications, INC). Cells were cultured at 37°C in a humidified atmosphere in the presence of 5% carbon dioxide and 95% air. Medium was changed every 3 days. Expansion was done by trypsinizing the cells (Trypsin/EDTA, Cell Applications, INC) and replating them when the proliferating cells had reached a sufficient density. Passaging was done at ratios of 1:6 (CnAOEC) or 1:3 (CnAOSMC). Cell counts were performed using the equipment Countess Automated Cell Counter (Invitrogen) following the manufacturer's instructions.

### Reagents and stimulation of CnAOEC and CnAOSMC

For stimulations, CnAOEC and CnAOSMC were grown for 4 days to obtain confluent cultures and were treated with 1 μg/ml of rDiACT or rDiFBAL, 10 μg/ml of PLG (Acris Antibodies) [4] or with a mixture of both treatments (rDiACT + PLG or rDiFBAL + PLG). Untreated cells and cells treated with rDiACT/rDiFBAL + PLG + 50 mM of εACA as an inhibitor of PLG activation were used as control cells under the same conditions.

### Cell lysates and Western blot analyses

Western blot analyses were performed as described in detailed previously [23] with slightly modifications. CnAOEC and CnAOSMC previously treated with 1μg/ml of rDiACT or rDiFBAL for 24 h were lysed in ice-cold lysis buffer (20mM Tris—HCl (pH 7.5), 140mM NaCl, 10mM ethylendiaminetetraacetic acid, 10% glycerol, 1% Igepal CA-630, aprotinin, pepstatin, and leupeptin at 1μg/ml each, 1mM phenylmethylsulfonyl fluoride, and 1mM sodium orthovanadate). Non-stimulated cells were used as controls under the same conditions. Protein samples (10 μg) were separated by SDS-PAGE under reducing conditions and electrotransferred onto polyvinylidine difluoride membranes. Then, membranes were blocked before incubation with the following primary rabbit polyclonal antibodies: anti-tPA and anti-uPA (Santa Cruz Biotechnology Inc) according to the manufacturer's recommendations. After incubation with HRP-conjugated anti-rabbit secondary antibodies, bands were visualized by a luminol-based detection system with p-iodophenol enhancement. Anti-α-tubulin antibody (Oncogene Research Products) was used as control to confirm loading of comparable amount of protein in each lane. Protein expression was quantified by densitometry using the PDQuest Software v.8.0.1 (Bio-Rad).

### Cell proliferation assay

Cells were plated on 24-well plates to a density of 10^4^ CnAOEC/well or 1.5 x 10^4^ CnAOSMC/well and allowed to attach overnight. After stimulations cell proliferation was analyzed by crystal violet nuclei staining over 10 days determining the number of viable cells as previously described [24]. Briefly, every two days, cells were rinsed with PBS, fixed with 4% formaldehyde for 10 min and stained with 0.2% crystal violet for 30 min at room temperature. After several rinses with PBS, the cells were allowed to dry overnight and crystal violet bound to cells was extracted by incubation with 2 ml/well of 10% acetic acid. The absorbance of the samples was then measured at 595 nm and transformed to “number of viable cells” using a curve that correlated absorbance and number of endothelial or smooth muscle cells previously determined.

### Cell migration assay

Cell migration was assessed by quantifying the percentage of wound closure in the wound-healing assay [25]. In brief, CnAOEC or CnAOSMC were cultured in 60 mm plates (3 x 10^5^ cells/plate) and allowed to attach overnight before wound creation. Confluent monolayers were wounded using a sterile pipette tip and the medium was exchanged for fresh medium before cell stimulation to remove cellular debris. The extent of wound closure of the treated and control cells was monitored over a time course by microscopy and determined along 8 hours by calculating the migrated distance/total wound distance.

### Collagen degradation assay

The concentration of collagen in the supernatants was analyzed by ELISA. Treated and control cells were cultured with medium for 48 hours. Then the culture supernatants were collected, filtered and added to multi-well plates (Costar). After incubation overnight at 4°C, wells were blocked with 1% BSA in PBS and incubated with a rabbit anti-Type I Collagen antibody (1:2500) (Acris Antibodies), and then with a peroxidase-conjugated goat anti-rabbit IgG (Sigma) at 1:500 dilution. All incubations were performed for 1 h at 37°C and between each step washed three times with PBST. Ortho-phenylene-diamine was used as a chromogen. ODs were measured at 492 nm in an Easy Reader (Bio-Rad).

### Matrix Metalloproteinase (MMP) levels assays

The levels of MMP-2 and MMP-9 metalloproteinases in the culture media of the different experimental groups was analyzed by gelatin zymography [26] according to the methodology described by Marangoni et al. (2011) [27]. Media samples employed in the collagen degradation assays were electrophoresed on a 10% polyacrylamide gel copolymerized with 1% gelatin (Sigma) together with a MMP marker (Cosmobio) as a positive control. The gels were washed for 1 hour in 2.5% Triton X-100 and incubated for 20 hours at 37°C in agitation in an enzymatic activation buffer ph 7.5 (50 mM Tris; 200 mM NaCl; 5 mM CaCl_2_; 0,2% Brij-35). The gels were finally stained with Coomassie blue. The positivity was assessed as appearance of clear bands on a dark background with molecular weights of 72 kDa (MMP-2) and 92 kDa (MMP-9). The levels of MMPs were calculated after measuring the intensity of the existing bands, which is directly proportional to the amount of gelatin degraded into the gel [28].

### Statistical analysis

The results from the PLG binding assay, PLG activation assay and Western blots for the tPA and uPA expression were analyzed with the Student’s t-test. Cell proliferation and migration, collagen degradation and MMP levels significance measurements (comparisons between groups) were performed by ANOVA and corrected for repeated measurements when appropriate. If ANOVA revealed overall significant differences, individual means were evaluated post hoc using Bonferroni’s procedure. All the results were expressed as the mean ± SD of three experiments performed with duplicates. In all experiments, a significant difference was defined as a p-value of <0.05 for a confidence level of 95%.

## Results

### Amplification, cloning, sequencing, and expression of *D*. *immitis* ACT and FBAL

Amplification of *D*. *immitis* ACT and FBAL cDNA by RT-PCR respectively resulted in PCR products of around 1100 and 1000 bp. After their cloning into the pSC-A vector, fragments were fully sequenced and their identities demonstrated as actin and fructose bisphosphate aldolase by BLAST analysis. The new sequences were respectively deposited in the Gen-Bank under accession numbers JQ780093.1 and JQ780094.1. The full cDNA respectively contained 1131 and 1092 nucleotides, encoded a protein of 376 and 363 amino acids, with a theoretical molecular weight of 41.820 and 39.423 Da, and pI of 5.29 and 7.65.

**Fig 1:**
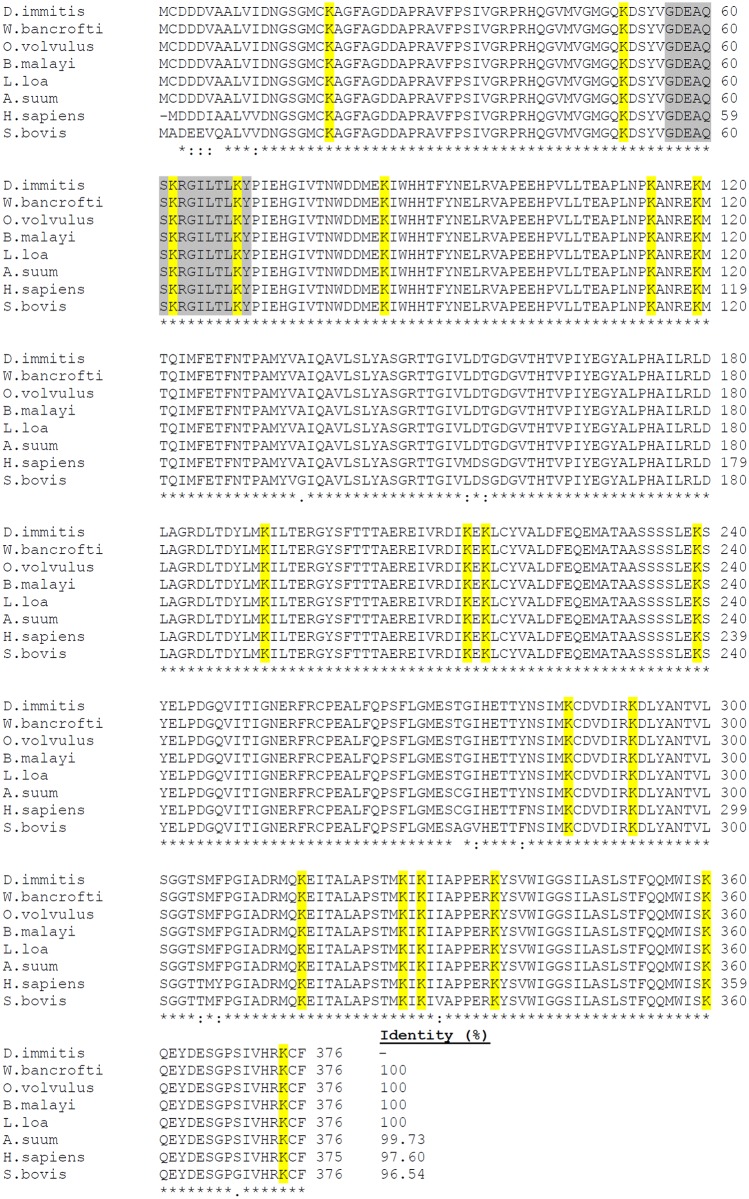
Multiple sequence alignment of DiACT with homologous proteins. Alignment of the *D*. *immitis* ACT sequence (I3WTW3) with the ACT from *W*. *bancrofti* (EJD75047), *O*. *volvulus* (EJW73626), *B*. *malayi* (P48812), *L*. *loa* (O01360), *Ascaris suum* (BAB68543), *Homo sapiens* (P20287) and *S*. *bovis* (AIE44418). The percentage of sequence identity between *D*. *immitis* sequence and the others is indicated. The amino acids conserved in all the sequences are labelled with asterisks, and conservative and semiconservative substitutions are respectively labelled with two and one point. Conserved lysine residues are shaded in yellow. The PLG-binding domain (GDEAQSKRGILTLKY) are shaded in grey.

**Fig 2:**
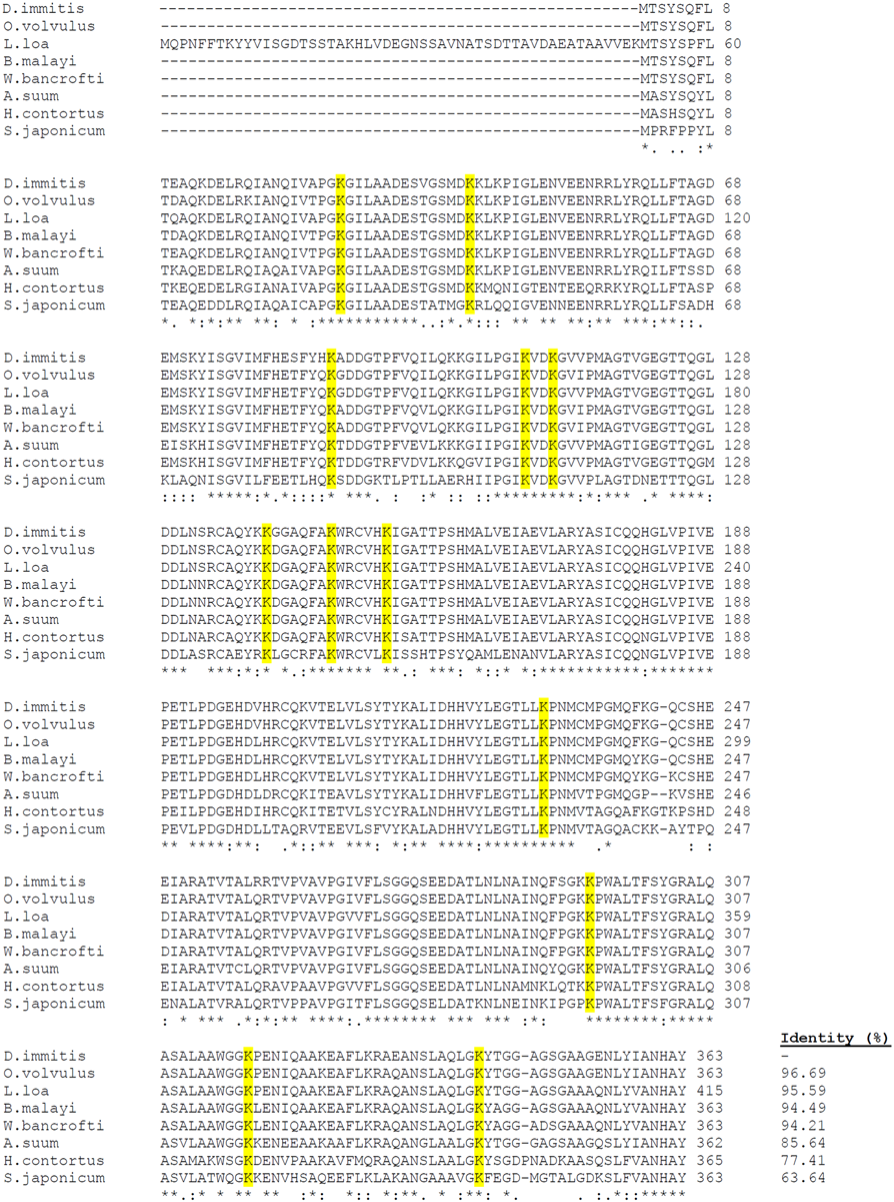
Multiple sequence alignment of DiFBAL with homologous proteins. Alignment of the *D*. *immitis* FBAL sequence (I3WTW4) with the FBAL from *O*. *volvulus* (Q9U9R9), *L*. *loa* (J0DRR2), *B*. *malayi* (A8P3ES), *W*. *bancrofti* (J9EVQ4), *A*. *suum* (U1M5S0), *Haemonchus contortus* (R4H2V1) and *S*. *japonicum* (C1LB95). The percentage of sequence identity between *D*. *immitis* sequence and the others is indicated. The amino acids conserved in all the sequences are labelled with asterisks, and conservative and semiconservative substitutions are respectively labelled with two and one point. Conserved lysine residues are shaded in yellow.

**Fig 3:**
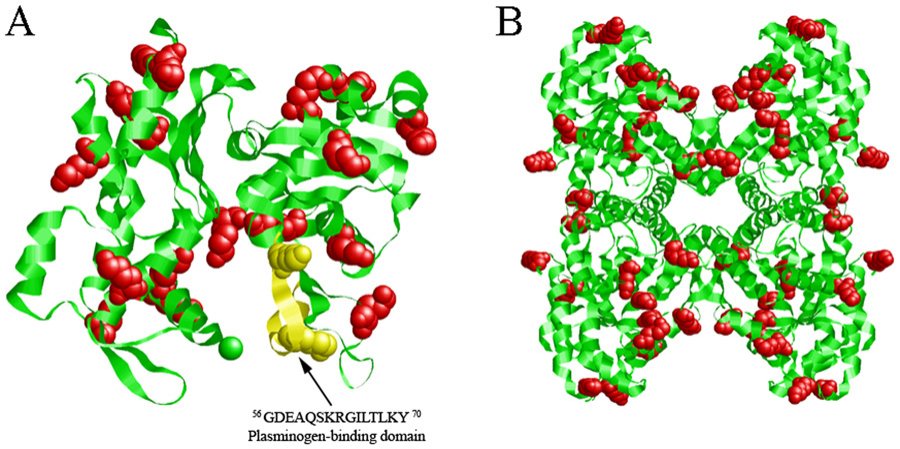
Molecular modelling of *D*. *immitis* ACT and FBAL. The secondary structure of the proteins was predicted with the Swiss-Model web server (http://swissmodel.expasy.org/) by analogy with the X-ray crystallography available models. The three-dimensional models of DiACT (A) and DiFBAL (B) were visualized with the RasMol application v. 2.7.5.2. Conserved lysine residues of proteins were highlighted as red balls. The PLG-binding domain (GDEAQSKRGILTLKY) is highlighted in yellow.

The bioinformatics analyses of the deduced amino acid sequences did not reveal a signal peptide, transmembrane helices or glycosyl-phosphatidyl inositol anchors. The percentage identity between DiACT/DiFBAL and homologous sequences from other organisms was analyzed using multiple sequence alignment with the ClustalW program (Figs 1 and 2). The analysis revealed that DiACT and DiFBAL are highly conserved proteins. Thus, in the alignment of these sequences with homologous proteins from other filarial nematodes (*Wuchereria bancrofti*, *O*. *volvulus*, *B*. *malayi* and *L*. *loa*) DiACT and DiFBAL respectively obtained identities of 100% and range of identities between 94.21 and 96.69%. These identities also obtained high values when DiACT and DiFBAL were aligned with homologous proteins from other parasitic helminthes (Figs 1 and 2). Aditionally, a PLG-binding domain to actin within amino acids 56 to 70 (GDEAQSKRGILTLKY) and 19 and 12 conserved lysine residues in the DiACT and DiFBAL alignment were respectively found and highlighted. In silico three-dimensional modelling of the molecules predicted the 3D structures showing in the case of DiACT a monomer with 20 α-helices and 19 β-sheets (Fig 3A). Molecular modelling of DiFBAL showing a homo-tetramer with the presence of 14 α-helices and 13 β-sheets (Fig 3B). The PLG-binding domain (GDEAQSKRGILTLKY) and the conserved lysine residues were highlighted and were visualized on the outside of the proteins.

The *D*. *immitis* ACT and FBAL cDNA were cloned into the expression vector TOPO/pDEST. After induction of expression in *E*. *coli*, the recombinant proteins were purified under denaturing conditions using nickel affinity chromatography. The purified rDiACT and rDiFBAL respectively had molecular weights of 43.6 kDa and 41.6 kDa in polyacrylamide gel.

### rDiACT and rDiFBAL bind PLG

**Fig 4:**
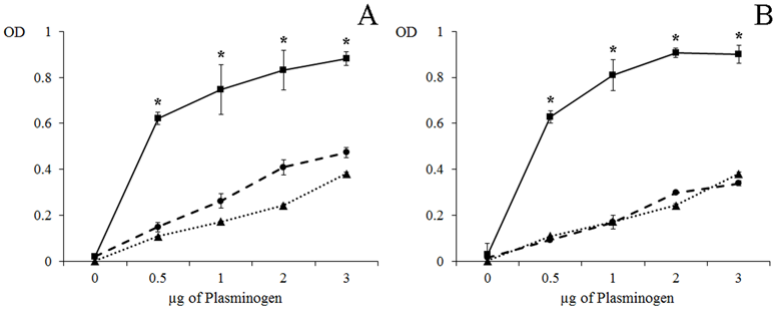
PLG binding assay of rDiACT and rDiFBAL. PLG binding to 0.5 μg of rDiACT (A) or rDiFBAL (B) analyzed over a range of PLG amounts using a microtiter plate method. (■) Incubation with increasing amounts of PLG, 0–3 μg. (▲) Competition assay with 50 mM εACA included during PLG incubation. (●) Wells coated with BSA used as negative control. Each point is the mean ± SD from three independent experiments. The asterisk (*) designates significant (p<0.05) differences.

An ELISA was carried out to determine the binding level of PLG to rDiACT and rDiFBAL (Fig 4). Analyses showed that both recombinant proteins bind PLG in a similar way and that this binding is directly proportional to the amount of PLG (Fig 4). Wells coated only with BSA, used as negative control, showed some non-specific binding activity, but always with values significantly lowers than those obtained by rDiACT and rDiFBAL (p<0.05). The addition of 50 mM εACA respectively resulted in the inhibition of about 70% and 90% of the binding level of rDiACT and rDiFBAL, demonstrating the involvement of lysine residues in the process. (Fig 4).

### rDiACT and rDiFBAL enhance the activation of PLG by tPA

**Fig 5:**
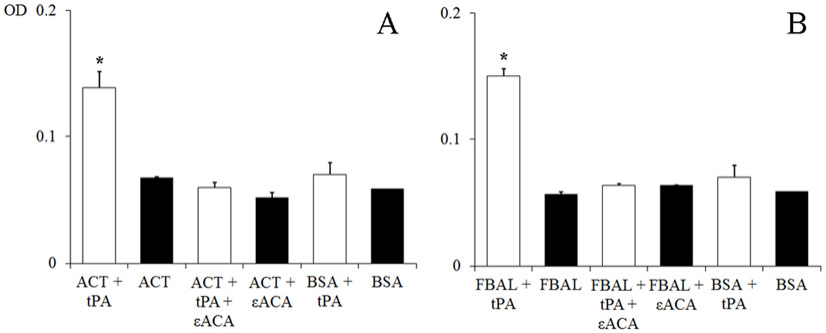
PLG activation assay of rDiACT and rDiFBAL. PLG activation and plasmin generation by rDiACT (A) or rDiFBAL (B). (□) 15 ng of tPA was added to mixtures which contained 2 μg of human PLG, 3 μg of D-Val-Leu-Lys 4-nitroanilide dihydrochloride (Sigma) and 1 μg of each recombinant protein (or BSA as negative control) in the presence or absence of 50 mM of εACA in a test volume of 100 μl. (■) Reaction mixtures in absence of tPA. Each point is the mean ± SD from three independent experiments. The asterisk (*) designates significant (p<0.05) differences.

In order to determine the ability of rDiACT and rDiFBAL to activate PLG and generate plasmin on their own, the amidolytic activity of plasmin generated in the presence or absence of tPA was measured. Negative controls replacing each recombinant protein for BSA or tPA were also used. Fig 5 shows the capacity of rDiGAPDH and rDiGAL to stimulate plasmin generation by tPA obtaining ODs significantly higher than the negative controls (p<0.05). Both proteins obtained similar results and PLG activation did not occur in the absence of tPA. In addition, this effect is inhibited by 50 mM εACA, indicating the involvement of lysine residues in the process (Fig 5).

### Effect of rDiACT and rDiFBAL on the fibrinolytic system activators (tPA and uPA) expression in CnAOEC and CnAOSMC

**Fig 6:**
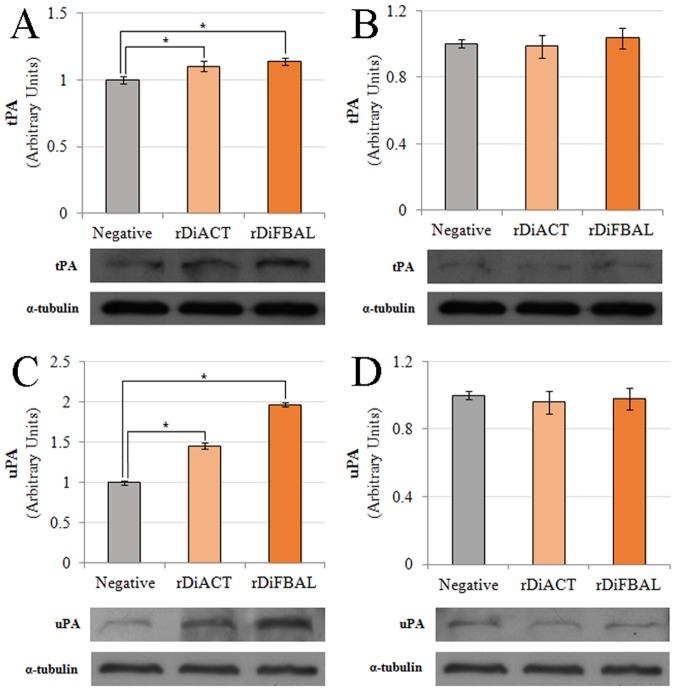
Effect of rDiACT and rDiFBAL on the fibrinolytic system activators expression. Effect of rDiACT and rDiFBAL on the expression of tPA (A and B) and uPA (C and D) in canine vascular endothelial (A and C) and smooth muscle cells (B and D). Protein extracts from lysed rDiACT or rDiFBAL treated or untreated confluent cell cultures were analyzed by Western blot for tPA and uPA. α-tubulin served as a protein control. Data are shown as representative images or means ± SD from three independent experiments. The asterisk (*) designates significant (p<0.05) differences from control cells. (■) Stimulated cells with 1μg/ml of rDiACT. (■) Stimulated cells with 1μg/ml of rDiFBAL. (■) Non-treated control cells.

To complete the study of the effect of rDiACT and rDiFBAL on the fibrinolytic system activation, proteins from rDiACT or rDiFBAL-treated or untreated CnAOEC and CnAOSMC extracts were separated by SDS—PAGE and analyzed by Western blotting using anti-tPA and anti-uPA antibodies. rDiACT and rDiFBAL induced a marked increase in the expression of the main fibrinolytic activators tPA and uPA in cultured endothelial cells (p<0.05) (Fig 6A and 6C). This increase was greater in the case of the uPA expression and significantly higher in cells stimulated with rDiFBAL (Fig 6C). Significant differences in the expression of tPA and uPA in CnAOSMC between rDiACT/rDiFBAL-treated or untreated cultures were not found (Fig 6B and 6D).

### Immunolocalization of DiACT and DiFBAL in sections from *D*. *immitis* adult worms

Immunolocalization of proteins was carried out by the use of a commercially available high-affinity ligand (in the case of ACT) and a rabbit polyclonal antisera (in the case of FBAL). The reactivity and specificity of this antiserum was tested in ELISA and Western blot prior to their use in the immunolocalization studies. The antibody titers of this antiserum were higher than 1/500, with an OD of 1.15 while the negative serum showed an OD of 0.13 at the same dilution. The anti-rDiFBAL antiserum reacted strongly and specifically against the recombinant protein, while the negative serum showed no reactivity in the Western blot analysis (not shown).

**Fig 7:**
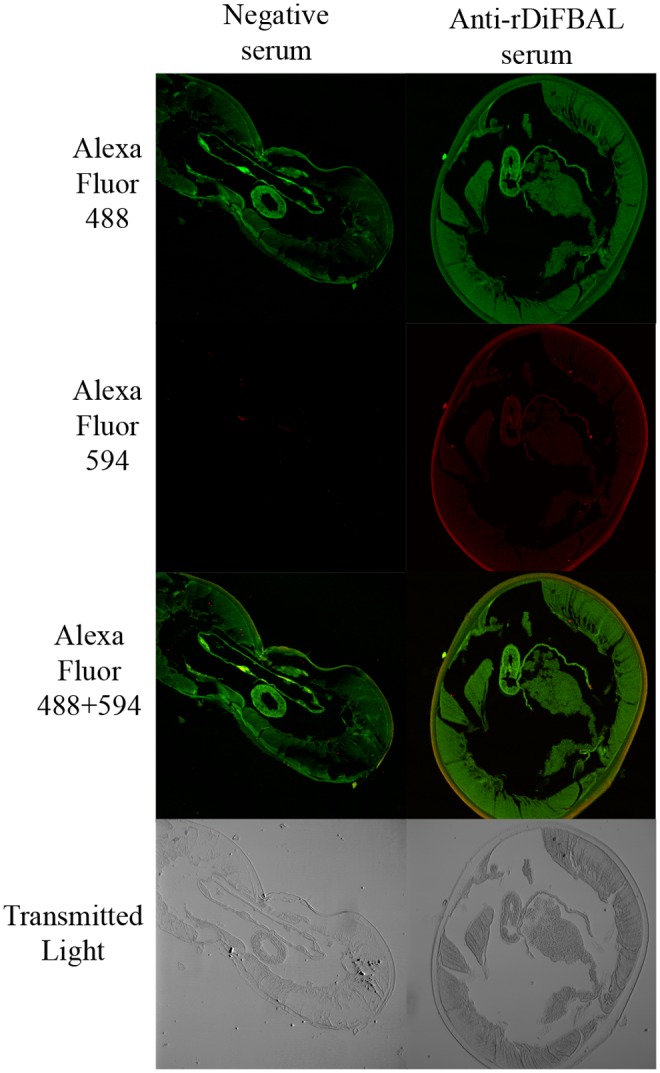
Immunolocalization of DiACT and DiFBAL in sections from *D*. *immitis* adult worms. Representative images from three independent experiments of parasite sections incubated with phalloidin-Alexa Fluor 488 (in green, specific binding to ACT) plus the negative or the anti-rDiFBAL rabbit sera and an anti-rabbit IgG-Alexa Fluor 594 (in red). Corresponding transmitted light images are also addressed. Magnification 4X.

The anatomical localization of DiACT and DiFBAL was carried out in histological sections of *D*. *immitis* adult worms by immunofluorescence. As shown in Fig 7, all sections showed green fluorescence throughout the soma of the parasite, as a result of the binding of phalloidin-Alexa Fluor 488, ACT high-affinity ligand which serves also as a positive control of the technique. Sections incubated with the anti-rDiFBAL antiserum showed, in addition, specific reactivity (in red) against the parasitic FBAL due to the binding of the anti-rabbit IgG antibody conjugated to Alexa Fluor 594. Both proteins are located scattered throughout all the soma, being especially abundant in the cuticle (reflected by an orange color in the overlay of Phalloidin-Alexa Fluor 488 + Alexa Fluor 594 images). Sections incubated with a rabbit negative serum showed no specific red fluorescence from recombinant proteins.

### rDiACT, but not rDiFBAL, produces proliferation of CnAOEC and CnAOSMC via PLG/plasmin system

**Fig 8:**
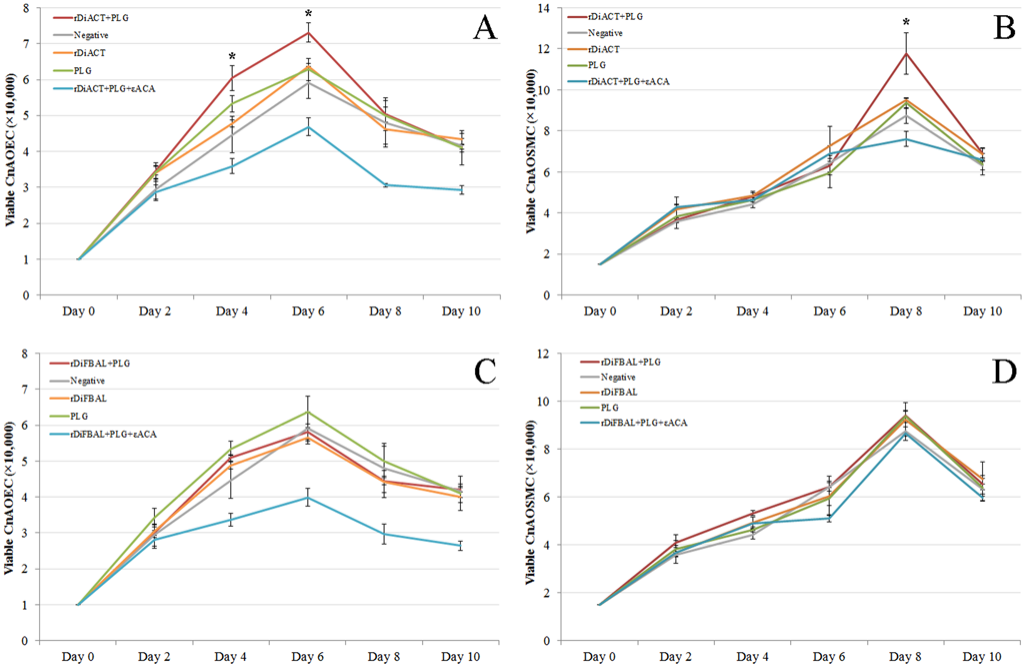
Cell proliferation assay performed by the crystal violet technique measuring cell viability over a 10 days period. The experiment was carried out in canine endothelial (A and C) and smooth muscle cells (B and D) untreated or treated with 1 μg/ml of rDiACT or rDiFBAL + 10 μg/ml of PLG, 1 μg/ml of rDiACT or rDiFBAL, 10 μg/ml of PLG, or with 1 μg/ml of rDiACT or rDiFBAL + 10 μg/ml of PLG + 50 mM of the εACA. Results were expressed as number of viable cells (x 10,000). Each point is the mean ± SD from three independent experiments. The asterisk (*) designates significant (p<0.05) differences between rDiACT + PLG treatment and control groups.

The effect of rDiACT or rDiFBAL and PLG on the proliferation of endothelial and smooth muscle cells was quantified using the crystal violet technique in a period of 10 days (Fig 8). Both cultures showed typical curves of cell growth in all experimental groups with a progressive growth between days 0 and 6 or 8 post-treatment in the CnAOEC or CnAOSMC cultures, experiencing cell death and an evident decrease of viable cells from there until the end of the experiment (day 10 post-treatment). Crystal violet staining showed an increase significantly greater in the number of viable cells in cultures stimulated with rDiACT + PLG than that showed by other experimental groups on days 4 and 6 post-treatment (CnAOEC) or day 8 post-treatment (CnAOSMC) (p<0.05), indicating that this treatment stimulates the proliferation of CnAOEC and CnAOSMC in culture. Significant differences in cell proliferation between cells stimulated with rDiFBAL + PLG and other experimental groups were not found in both types of cultures.

### rDiACT and rDiFBAL produce migration of CnAOEC and CnAOSMC via PLG/plasmin system

**Fig 9:**
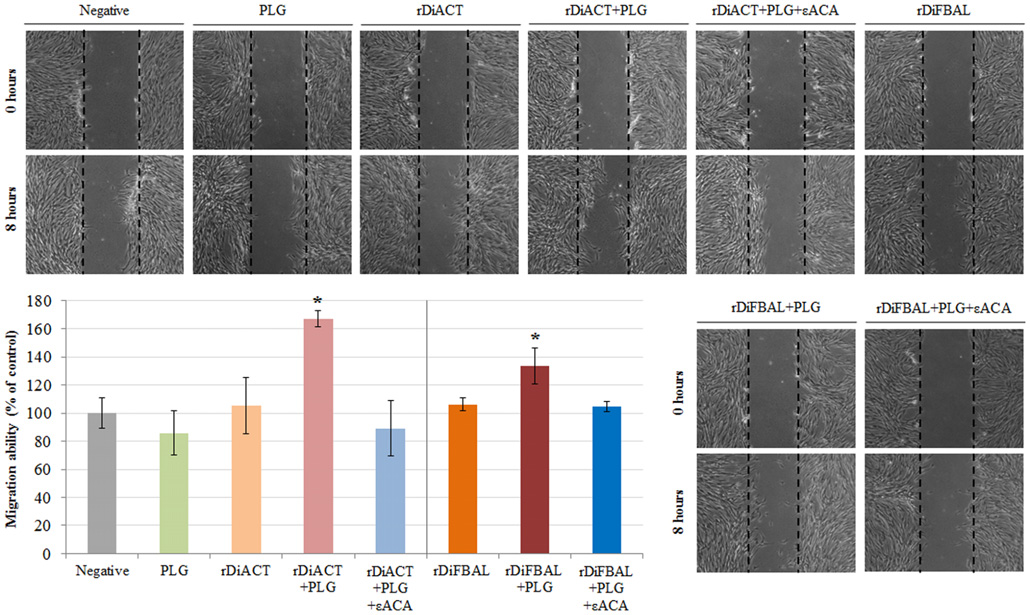
CnAOEC migration by Wound-Healing assay. Confluent cell cultures were wounded post-treatment and migration distances were measured at 8 hours. The experiment was carried out in canine endothelial cells untreated or treated with 10 μg/ml of PLG, 1 μg/ml of rDiACT/rDiFBAL, 1 μg/ml of rDiACT/rDiFBAL + 10 μg/ml of PLG or with 1 μg/ml of rDiACT/rDiFBAL + 10 μg/ml of PLG + 50 mM of the εACA. The results were expressed as percentage of the migration ability of the negative control cells (100%). Data are shown as representative images or means ± SD from three independent experiments. The asterisk (*) designates significant (p<0.05) differences between rDiACT or rDiFBAL + PLG treatment and control groups.

**Fig 10:**
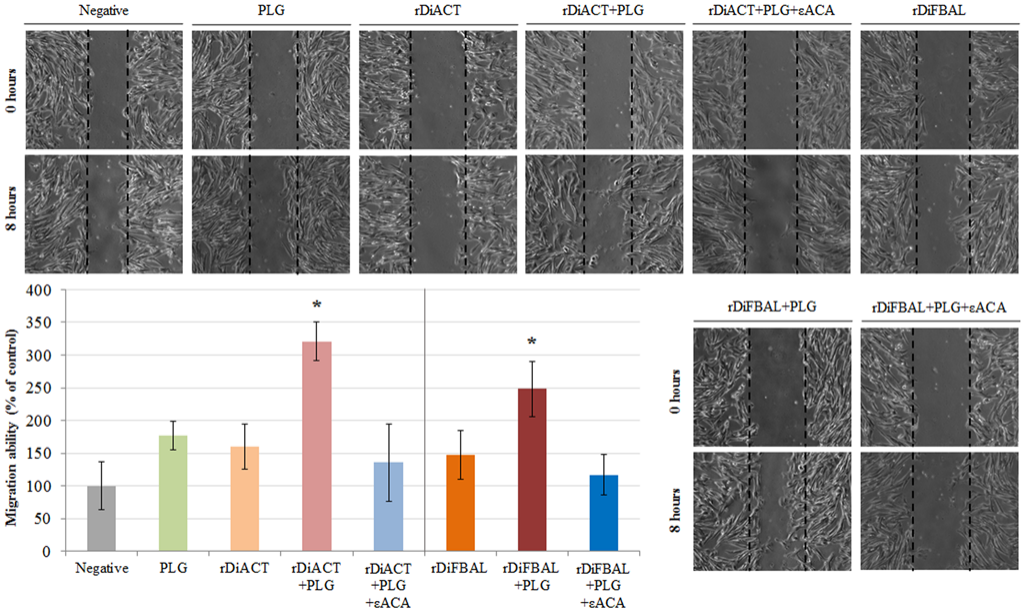
CnAOSMC migration by Wound-Healing assay. Confluent cell cultures were wounded post-treatment and migration distances were measured at 8 hours. The experiment was carried out in canine smooth muscle cells untreated or treated with 10 μg/ml of PLG, 1 μg/ml of rDiACT/rDiFBAL, 1 μg/ml of rDiACT/rDiFBAL + 10 μg/ml of PLG or with 1 μg/ml of rDiACT/rDiFBAL + 10 μg/ml of PLG + 50 mM of the εACA. The results were expressed as percentage of the migration ability of the negative control cells (100%). Data are shown as representative images or means ± SD from three independent experiments. The asterisk (*) designates significant (p<0.05) differences between rDiACT or rDiFBAL + PLG treatment and control groups.

A Wound Healing assay was performed to assess migration of endothelial (Fig 9) and smooth muscle cells (Fig 10). The quantification was carried out by measuring the distance of migration in comparison with negative control (untreated cells) to 8 hours post-treatment. In both CnAOEC and CnAOSMC cultures a significant increase of cell migration after stimulation with rDiACT or rDiFBAL + PLG with respect to the other experimental groups (p<0.05) occurred, being this increase most pronounced in cultured smooth muscle cells. After comparing the effect of both parasitic proteins, rDiACT showed higher values of migration ability in both types of cell cultures with respect to those obtained by rDiFBAL.

### rDiACT and rDiFBAL produce ECM degradation of CnAOEC and CnAOSMC via PLG/plasmin system

**Fig 11:**
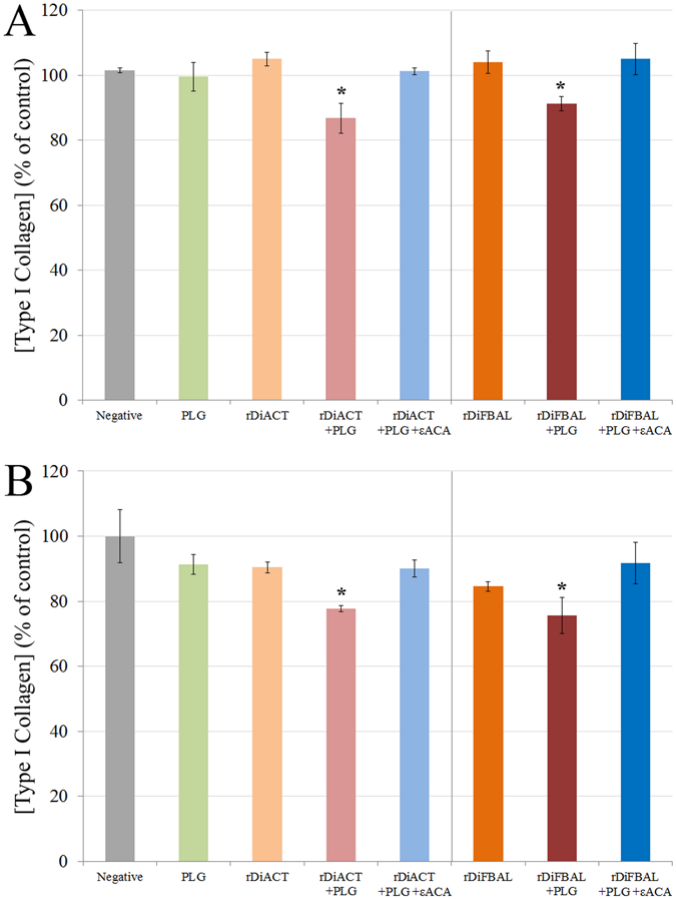
Type I Collagen degradation assay. Collagen degradation measured in culture supernatants from canine endothelial (A) and smooth muscle cells (B) untreated or treated with 10 μg/ml of PLG, 1 μg/ml of rDiACT/rDiFBAL, 1 μg/ml of rDiACT/rDiFBAL + 10 μg/ml of PLG or with 1 μg/ml of rDiACT/rDiFBAL + 10 μg/ml of PLG + 50 mM of the εACA. The results were expressed as percentage of the Type I Collagen concentration in the culture supernatant from negative control cells (100%). Each point is the mean ± SD from three independent experiments. The asterisk (*) designates significant (p<0.05) differences between rDiACT or rDiFBAL + PLG treatment and control groups.

**Fig 12:**
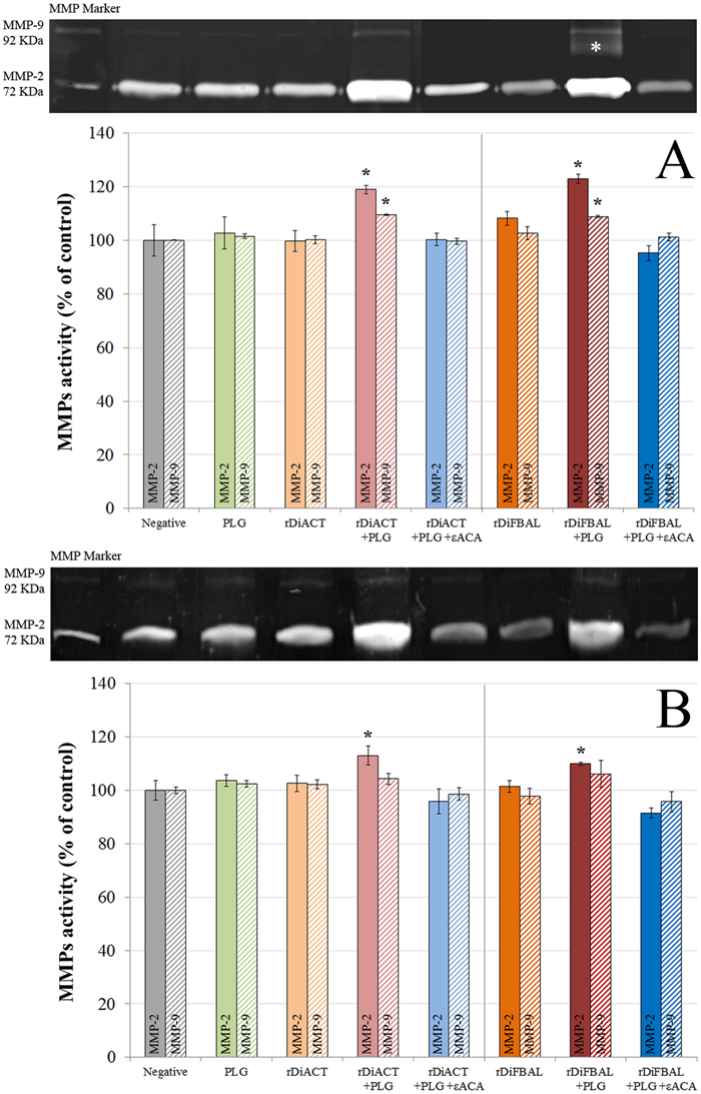
MMP-2 and 9 levels assay. Representative zymography of MMP-2 (solid bars) and MMP-9 (hatched bars) levels in the culture supernatants from canine endothelial (A) and smooth muscle cells (B) untreated or treated with 10 μg/ml of PLG, 1 μg/ml of rDiACT/rDiFBAL, 1 μg/ml of rDiACT/rDiFBAL + 10 μg/ml of PLG or with 1 μg/ml of rDiACT/rDiFBAL + 10 μg/ml of PLG + 50 mM of the εACA. Note the gelatinolytic bands associated with MMP-2 (72 KDa) and MMP-9 (92 KDa) levels, as well as with the MMP-9 activated form (marked with a white asterisk at 82KDa. The results were expressed as percentage of the MMPs levels in the culture supernatant from negative control cells (100%). Data are shown as representative images or means ± SD from three independent experiments. The asterisk (*) designates significant (p<0.05) differences between rDiACT or rDiFBAL treatment and control groups.

To examine ECM degradation, Type I Collagen in the culture supernatant of treated and untreated CnAOEC and CnAOSMC were measured by ELISA (Fig 11). A lower concentration of Type I Collagen and therefore a further degradation of the secreted collagen by the cells was observed in the culture media of CnAOEC and CnAOSMC stimulated with rDiACT/rDiFBAL + PLG than that obtained by the control cells (p<0.05). In addition, the same culture media from treated and untreated cells were analyzed with gelatin zymography for MMP-2 and MMP-9 levels (Fig 12). Density of the bands was measured by the Quantity One Software (Bio-Rad). Treatment with rDiACT or rDiFBAL + PLG shows a significantly higher MMP-2 level in the CnAOEC and CnAOSMC culture media and MMP-9 level in the CnAOEC culture media than that obtained by the other treatments (p<0.05). In addition, treatment with rDiFBAL + PLG shows an activation of the latent form of the MMP-9 in the CnAOSMC culture media (show by a clear band of 82 kDa), which does not appear with other treatments. No significant differences in the MMP-9 levels in the culture media of CnAOSMC were observed (Fig 12).

## Discussion

The main finding of this study lies in relating how the activation of the fibrinolytic system by two proteins of the blood-borne parasite *D*. *immitis* (a priori beneficial for both the parasite and host), may cause long-term pathological effects based on the participation of generated plasmin in the emergence of a process of proliferative endarteritis. This study was conducted with the ACT and FBAL of *D*. *immitis*, highly conserved proteins that were selected among parasite antigens that had been identified as PLG-binding proteins in previous works [17,18]. Both proteins have previously been linked to pro-fibrinolytic activities. The interaction between actin and PLG is well known, as well as the fact that specific binding occurs through lysine residues, which stimulate the tPA-dependent plasmin generation [29]. In addition, its function as PLG receptor has been demonstrated on the surface of endothelial cells [30] and in the tegument of *S*. *bovis* [31]. Meanwhile, FBAL has been identified as PLG-binding protein in the bacterium *Mycobacterium tuberculosis* [32], the fungal pathogens *Candida albicans* [33] and *Cryptococcus neoformans* [34] and in the helminth parasite *S*. *bovis* [31].

In this paper, two peptide sequences of 376 and 363 amino acids were respectively obtained by cloning and sequencing of the *D*. *immitis* ACT and FBAL cDNAs. The subsequent bioinformatic analyses based on the multiple sequence alignments carried out with homologous proteins from other helminth parasites and the homology modelling of their 3D structures have highlighted their high degree of conservation. None of the proteins showed structural motifs for their transport or expression on the cell surface (signal peptide, transmembrane motifs or GPI anchors), despite the fact that both proteins have been previously identified in the secretome and on the surface of *D*. *immitis* [18,35]. This may be related to unconventional mechanisms of protein transport, as for example with the association of these proteins with exosome-like secretion vesicles. This fact has been recently postulated as an extracellular transport mechanism for glycolytic enzymes from several groups of parasites [36].

In order to assess the interaction of these proteins with the host fibrinolytic system we study their abilities to bind PLG, enhance plasmin generation, stimulate the production of the fibrinolytic activators tPA and uPA, as well as their locations in the adult parasite. Both proteins rDiACT and rDiFBAL showed ability to bind PLG and stimulate plasmin generation by tPA, which are capabilities mediated by the participation of lysine residues, as it has been demonstrated by competition assays carried out with εACA. Interaction with PLG has been historically associated with the presence of carboxyl-terminal lysine residues in their receptors [37]. However, conserved internal lysine residues have been more recently described as PLG-binding domains as in the case of enolase of *Streptococcus pneumoniae* [38] or human beta-actin, in which a PLG-binding domain within amino acids 55 to 69 (GDEAQSKRGILTLKY) has been identified indicating that Lys^61^ and Lys^68^ are essential for this action [39]. This domain has been conserved in the ACT of *D*. *immitis* (see Fig 1). In addition, after viewing the spatial location of the conserved lysine residues of the DiACT and DiFBAL in their 3D models, these residues seem to be located externally in these molecules, which would facilitate the accessibility of PLG.

Despite the fact that the generation of plasmin by rDiACT and rDiFBAL is dependent on tPA availability, we demonstrate that both proteins produce a significant stimulation of the basal production not only of this fibrinolytic activator, but also of uPA in canine endothelial cells in culture. This result reinforces the pro-fibrinolytic condition of these proteins, since the participation of tPA and uPA in fibrinolysis is essential in the effective activation of PLG [40]. On the other hand, high levels in the expression of both tPA and uPA have been related to several physiological and pathological processes like tissue remodeling and chronic inflammatory diseases, such as atherosclerosis and arthritis [41,42]. Finally, immunolocalization studies showed that DiACT and DiFBAL, as well as having an intracellular location, they are particularly abundant in the cuticle of *D*. *immitis*. This fact is essential, so that the interaction of these proteins with the fibrinolytic system may have relevance in vivo, it is necessary that DiACT and DiFBAL are expressed in tissues in direct contact with the blood of the host [43].

Secondly, in order to study the effect of plasmin resulting from the fibrinolytic activation by DiACT and DiFBAL on the proliferative endarteritis in the canine arterial wall, we have developed an “in vitro” model of canine endothelial and smooth muscle cells. Our data demonstrate that stimulation with rDiACT + PLG causes the proliferation of CnAOEC and CnAOSMC, and treatments with rDiACT or rDiFBAL + PLG enhance migration of both types of cells. This would be consistent with the formation of intravascular microvilli occurring during dirofilariosis, which is result of the multiplication and migration of the arterial wall cells [44]. In addition, the binding of both proteins to PLG causes a significant increase in the degradation of collagen type I and in the levels of MMP-2 in the culture media of CnAOEC and CnAOSMC, as well as in the levels of MMP-9 in the culture media of CnAOEC. Moreover, the binding of rDiFBAL and PLG seems to induce an activation of the latent form of the MMP-9 in the culture media of CnAOSMC. These facts highlight the participation of the rDiACT/rDiFBAL + PLG interaction in the degradation of the ECM needed for the formation of intravascular microvilli. Type I Collagen represents the main component of the ECM of elastic arteries. Its alteration has been associated with vascular disease and its degradation products with the proliferation and migration of smooth muscle cells in remodeling arteries [45]. These results are consistent with those observed in vivo by Wang et al. (2005) who reported a significantly lower amount of collagen in heartworm-infected dogs than in clinically normal dogs [15]. On the other hand, MMPs function in the extracellular environment of cells and degrade ECM molecules from the tissue. Among them, Gelatinases (MMP-2 and MMP-9) can digest a large number of the ECM molecules including type IV, V and XI collagens, laminin, aggrecan core protein, etc. MMP-2, but not MMP-9, also digests collagens I, II and III [46]. In addition, the pathophysiological study of the action of gelatinases shows that an increase in its activity can be responsible for tissue remodeling, hypertrophy, angiogenesis and chronic inflammation [47]. Finally, inhibition of all positive results by including the 50 mM εACA in the stimulations demonstrates the final participation of plasmin generated by binding between DiACT or DiFBAL and PLG on the proliferation and migration of CnAOEC and CnAOSMC, as well as the degradation of the ECM.

These results seem to indicate that *D*. *immitis* could use DiACT and DiFBAL to shift the fibrinolytic balance towards the generation of plasmin, which might constitute a survival mechanism to avoid the clot formation in its intravascular habitat. On the other hand, in long-term infections as cardiopulmonary dirofilariasis, this overproduction of plasmin could be related to pathological phenomena described in the emergence of proliferative endarteritis. These findings contribute to understand a very complex part of the host-pathogen relationships of dirofilariasis, showing how a process related to the survival of the parasite and the host can lead to a pathogenic mechanism of great importance. Since the ability to bind PLG and enhance plasmin generation of proteins of many pathogens has been shown, and that ACT and FBAL are highly conserved pathogenic antigens, similar events could occur in other infections caused by vascular pathogens developing chronic processes. The knowledge of these mechanisms could be critical for the treatment and prevention of diseases caused by infectious agents.
